# Interprofessional Education and Collaboration in Healthcare: An Exploratory Study of the Perspectives of Medical Students in the United States

**DOI:** 10.3390/healthcare7040117

**Published:** 2019-10-15

**Authors:** Sunitha Zechariah, Benjamin E. Ansa, Stephanie W. Johnson, Amy M. Gates, Gianluca De Leo

**Affiliations:** 1 Applied Health Sciences Program, College of Allied Health Sciences, Augusta University, Augusta, GA 30912, USA; sjohnson8@augusta.edu (S.W.J.); amgates@augusta.edu (A.M.G.); gdeleo@augusta.edu (G.D.L.); 2 Morrison Healthcare, Sandy Springs, GA 30350, USA; 3 Institute of Public and Preventive Health, Augusta University, Augusta, GA 30912, USA; 4 Occupational Therapy, College of Allied Health Science, Augusta University, Augusta, GA 30912, USA; 5 Neonatal Intensive Care Unit, Medical College of Georgia, Augusta University, Augusta, GA 30912, USA; 6 Dept. of Interdisciplinary Health Sciences, College of Allied Health Sciences, Augusta University, Augusta, GA 30921, USA

**Keywords:** interprofessional, education, collaboration, healthcare, medical students, attitudes, behaviors, perceptions

## Abstract

Qualified and competent healthcare professionals working in a collaborative team environment is a prerequisite for high quality patient care. In order to be successful in the healthcare working environment, medical students need to be exposed to interprofessional learning early in their education. A single stage online survey was administered to medical students to evaluate their attitudes and perceptions of interprofessional education (IPE) and whether prior exposure to IPE increased their appreciation for interprofessional collaboration. The results suggest that irrespective of prior exposure to IPE, medical students appreciated the importance of interprofessional education and collaboration. Medical students showed a strong interest in attending interprofessional courses in other disciplines. Time constraints, scheduling conflicts, and communication emerged as barriers to IPE. Medical students embraced IPE and welcomed the opportunity to learn with other disciplines. Clinical case studies and simulations were identified as potential methods to integrate with other healthcare disciplines. The positive attitude and perceptions of the medical students toward interprofessional education and collaboration warrants the inclusion of related courses in medical curricula, as this may further increase students’ potentials in becoming effective healthcare providers.

## 1. Introduction

Interprofessional Education (IPE), an essential pedagogical approach in healthcare education, is deemed crucial in equipping healthcare professionals to deliver safe, high quality, and optimal patient care [1]. IPE is the engagement of two or more healthcare professions in an integrated learning environment in an effort to foster collaboration and improve health [2]. IPE fosters interprofessional collaboration (IPC), which is often recognized for nurturing a collaborative team approach, resulting in an improved quality of patient care, decreased length of hospital stay, reduced costs of care, and fewer medical errors [3]. Additionally, a systematic review by Reeves et al. [4] showed improved patient outcomes, better clinical processes, and enhanced patient satisfaction when IPC is utilized.

IPC, on the other hand, is a process where multiple individuals from diverse backgrounds collaborate to deliver the best possible care to their patients [2]. Wheelan et al. [5] noted that improved healthcare outcomes for intensive care unit patients is dependent upon teamwork and collaboration among healthcare providers. The Committee on Quality Health Care in America [6] stated that providing opportunities for interdisciplinary training is vital for reinventing healthcare, with an increased focus on evidence-based practice. Asmirajanti et al. [7] reviewed clinical care pathways with IPC and found that while clinical care pathways need active engagement and collaboration to yield positive results from healthcare providers, the optimization of care needs to be implemented within the realm of a multidisciplinary team. To achieve such well-functioning, collaborative interprofessional teams, it is fundamental to expose students to interprofessional learning during their medical training [8]. Embedding a strong IPE foundation and interprofessional learning opportunities within healthcare education is therefore paramount, as has long been emphasized by the World Health Organization (WHO) [9,10], specifically for medical students [11,12,13]. IPE is indispensable for medical students, as they typically assume the lead role in patient care when they commence their medical practice. This level of responsibility requires medical students to function with the necessary team and communication skills, along with a healthy respect for their fellow healthcare team members [14].

Although IPE is not a new concept in the medical profession, it has gained momentum recently, as the Association of American Medical Colleges (AAMC) and Institute of Medicine (IOM) have endorsed it to be an integral component of medical education, with the AAMC listing it as one of its strategic initiatives in medical training [15]. Harden [14] recommends introducing IPE in medical education early in the training. His review rationalizes that the early introduction of IPE will shape young minds and instill the appropriate attitude toward other healthcare professionals before any biases are developed. Despite the mounting needs for IPE in the medical curriculum, only 38% of surveyed medical schools within the United States offer it as part of their formal or informal curricula [16], demonstrating inadequacies in IPE provision and the training of medical students. One possible challenge encountered in incorporating IPE in medical and other health professions’ educational curricula is the lack of structured competencies integrating various disciplines. The IPEC Expert Panel addressed this concern and developed four core interprofessional competencies, i.e., ethics, communication, roles and responsibilities, and teams/teamwork, to enhance collaboration among healthcare professionals in order to improve health outcomes [8].

The extent of IPE offered in medical education and the receptiveness of medical students to engage in interprofessional learning are well known to directly influence their eagerness to participate in IPC and assimilation [17]. A longitudinal study conducted at a private university in the United States explored student attitudes toward IPE [18]. This study surveyed medical, nursing, and physician assistant (PA) students in their first year of training and again in their third year. The results demonstrated that medical students had a less positive attitude toward IPE compared to nursing and PA students. Conversely, Ahmed and colleagues [19] report a high level of IPE readiness among their first year medical students at the National University of Singapore compared to their first year nursing, pharmacy, and dentistry students. The differences noted in these studies invoke the need to explore the perceptions of medical students. Hence, this study aimed to evaluate the attitudes and perceptions of a sample of medical students undergoing training in the United States toward IPE and IPC in healthcare teamwork and patient care.

## 2. Materials and Methods

### 2.1. Study Population

Study participants were recruited in partnership with the American Medical Student Association (AMSA) [20]. AMSA is a United States-based, independent membership organization consisting of medical and premedical students, interns, and residents. Only medical students trained within the United States and listed in the AMSA registry were included in this study.

### 2.2. Survey Design

A 34-item, single-stage survey was developed utilizing previously published instruments [21,22,23] along with survey questions created by the authors to tailor to medical students. Questions included empirical, Likert scale, and qualitative/open-ended questions. The survey was designed through the Qualtrics platform (Qualtrics, Provo, UT); it consisted of three sections: (1) Basic demographic information (age, gender, race, year of medical school), (2) attitudes and perceptions regarding interprofessional education and collaboration, and (3) qualitative/open-ended responses regarding barriers and opportunities for IPE.

In addition, the survey tested the medical students’ general IPE knowledge. Using the WHO’s definition of IPE [2], the participants were provided with an option to agree or disagree with the definition based on their knowledge level. Participants were then asked to rank the four core IPEC competencies (Ethics, Communication, Roles/Responsibilities, and Teams and Teamwork) in the order of importance, with 1 being the most important and 4 the least. Each ranking order could only be assigned once. To measure changes in understanding or opinion that may have developed as the survey progressed, the definitions for each of the four core IPEC competencies were provided at the end of the survey, and the students were asked to rank the competencies again in order to identify any changes in their views.

### 2.3. Data Collection

The anonymous survey link generated from Qualtrics was emailed electronically by AMSA to medical students receiving training in the United States, and included in AMSA membership database (*n* = 7771). The survey remained available for 6 weeks from the initial email distribution. Unfortunately, AMSA was unable to send reminder emails during the study period.

### 2.4. Statistical Analysis

The statistical data analysis was conducted using SPSS version 25 (IBM SPSS, Inc., Armonk, NY, USA). Descriptive statistics were utilized to examine the medical students’ current IPE exposure. Medical students who received IPE within their curriculum as a core component, as an elective, or both, were coded together and categorized as ‘IPE received’, while those who never received any form of IPE within their curriculum were categorized as ‘No IPE received’. The frequency of responses to questions about attitudes and perceptions toward IPE coursework, IPC in patient care, and IPC toward healthcare team, were calculated. An independent samples t-test was performed to determine statistically significant differences between the means of attitudes and perceptions of medical students who received IPE and those who never received it within their program. Scores were assigned to each of the Likert Scale ratings with a range of ‘1’ to ‘5’. “Strongly disagree” was scored as ‘1’ and “strongly agree” was scored as ‘5’. Statistical significance was set at the level of *p* < 0.05. TagCrowd (San Francisco, CA, USA), an online application for creating word clouds, was used to visualize open-ended responses to questions regarding barriers and opportunities for IPE.

### 2.5. Ethical Considerations

The Institutional Review Board at Augusta University approved this study (IRB# 1151240–2).

## 3. Results

### 3.1. Demographics

A total of 98 medical students responded to the survey. Only surveys completed to their entirety (*n* = 69) were included in the analyses. Table 1 shows the demographic distribution of the survey respondents.

The majority of respondents were 20–29 years old (*n* = 59, 85.5%), predominantly male (*n* = 42, 61%), and white (*n* = 48, 69.5%). A slightly higher percentage of respondents were year 3 medical students (*n* = 22, 31.8%), followed by year 1 students (*n* = 17, 24.6%), and an equal percentage of year 2 and year 4 medical students (*n* = 15, 21.7%). A large number of respondents (*n* = 52, 75.4%) reported receiving IPE infused within their curriculum as a core component, as an elective, or both. Almost one in every four subjects (*n* = 17, 24.6%) indicated not receiving any form of IPE within their curriculum.

The total number of credit hours received varied among the medical students who had received IPE within their curriculum. Among these, 19 students (25.7%) received one credit; 9 (13%) received two credits; 10 (14.5%) received three credits, and 14 (20.3%) received four credit hours. Of those who were offered IPE, 31 students (44.9%) agreed that the amount of IPE offered in their curriculum promoted interprofessional collaboration and practice.

Among the medical students who received IPE, a majority (*n* = 42, 80.7%) reported being engaged with nursing students compared to any other disciplines, including advanced practice nurses/nurse practitioners, dietitians/nutritionists, occupational therapists, pharmacists, physical therapists, physician assistant, respiratory therapists, social workers, and/or speech-language pathologists. The least engaged discipline reported was respiratory therapy (*n* = 6, 11.5%).

Clinical simulation was found to be the preferred IPE method of learning (*n* = 43, 82.7%) among the medical students who had received IPE, followed by informal clinical experience (*n* = 36, 69.2%) when compared to other options including case studies, integrated classes, core and elective coursework, optional conferences, and training.

Of those who reported never receiving IPE, 16 students (94%) desired IPE to be part of their course curriculum, of which 10 (59%) preferred a core course while 6 (35%) preferred optional training. Clinical simulation was also the preferred method of IPE among those who had never received IPE (*n* = 14, 82.3%), followed by multidisciplinary conferences as their second choice of learning method (*n* = 11, 65%). Among the medical students who never received IPE, 7 (41.1%) favored a curriculum that included IPE four or more times to promote interprofessional collaboration and practice.

### 3.2. Attitudes

The attitudes of the medical students toward interprofessional education and collaboration was examined to detect any differences between having prior exposure to IPE within their medical curriculum compared to no previous exposure to IPE. Each of the individual items demonstrating their attitudes and perceptions toward IPE coursework, IPC in patient care, and IPC with a healthcare team is discussed in detail below and is presented in Table 2a–c.

#### 3.2.1. Attitudes Toward IPE Coursework

A majority of the medical students indicated a strong interest in attending IPE coursework with other disciplines (*n* = 57, 82.6%, strongly agreed and agreed combined). There was strong consensus on attending the IPE coursework with nursing students, i.e., *n* = 61, 88.4% either agreed or strongly agreed, followed by allied health professionals (*n* = 57, 82.6%), and nursing practitioners and physician assistants (*n* = 53, 76.8%). About 77% (*n* = 53) strongly agreed or agreed that medical students would benefit from working on group projects with students from other healthcare professions. Nearly 90% (*n* = 62, 89.9%) of medical students agreed that IPE with other disciplines improves interprofessional communication. Similarly, 84% (*n* = 58) agreed that integrated learning with students from other disciplines helps medical students to become effective healthcare team members. There was a high level of agreement (*n* = 66, 95.7% strongly agreed and agreed) among the medical students that interprofessional learning would aid in understanding their own professional limitations. As expected, medical students strongly disagreed or disagreed (*n* = 40, 52.1%) that clinical problem solving could only be learned effectively when taught within their individual department or school. However, a majority remained neutral or disagreed (*n* = 54, 78.2%) that healthcare students require interprofessional learning to increase their ability in understanding clinical problems.

#### 3.2.2. Attitudes Toward IPC in Patient Care

Exploring the IPC patient care attitudes of medical students who had prior exposure to IPE compared to those who never received IPE showed that the medical students strongly agreed (*n* = 60, 87%, strongly agreed and agreed combined) that IPC increases the healthcare team’s ability to understand clinical problems. Similarly, there was a high level of agreement that the interprofessional approach to patient care is more efficient, i.e., *n* = 58, 84.1% strongly agreed and agreed, and results in treating patients as a whole person (*n* = 56, 81.2% strongly agreed and agreed). A strong agreement, almost reaching statistical significance (*p* = 0.05), was evident for patients ultimately benefitting if healthcare professionals worked together to solve patient problems (*n* = 66, 95.7%, strongly agreed and agreed). Equally, a solid level of agreement was seen for the ‘give and take’ among team members helping providers make better patient care decisions (*n* = 60, 87%, strongly agreed and agreed). Strong agreement was also indicated for team members understanding the roles of other healthcare professionals when observations were exchanged as a multi-disciplinary team (*n* = 64, 92.8%, strongly agreed and agreed). There were no statistically significant differences in attitudes toward IPC for patient care, irrespective of whether they had previous IPE exposure.

#### 3.2.3. Attitudes Toward IPC with Healthcare Team

Analyzing the IPC healthcare team attitudes and perceptions of medical students who had prior exposure to IPE compared to those who had never received IPE revealed a statistically significant difference (*p* = 0.003) in their belief that healthcare teams need to trust and respect each other for effective interprofessional collaboration (*n* = 67, 97.1%, strongly agreed and agreed). There were no other statistically significant differences noted; however, positive agreements were displayed for all areas of IPC attitudes toward healthcare team. Medical students strongly agreed that IPC helps healthcare professionals to think positively about the healthcare team (*n* = 57, 82.6%, strongly agreed and agreed). Equally strong agreement was seen with communication skills being critical for the healthcare team for improved patient outcomes (*n* = 69, 95.7%, strongly agreed and agreed). Medical students also agreed that IPC allows healthcare professionals to understand their role limitations (*n* = 63, 91.3%, strongly agreed and agreed) and that team meetings foster communication among members of different disciplines (*n* = 63, 87%), strongly agreed and agreed). Responses for the question ‘working in an interprofessional environment keeps health professionals enthusiastic and interested in their jobs’ varied, with 60.9% agreeing (*n* = 42) and 34.7% (*n* = 24) disagreeing with this statement. Similarly, mixed responses were seen for ‘working in an interprofessional manner requires additional time’ (*n* = 36, 52.2%, strongly agreed and agreed & *n* = 33, 47.8%, strongly disagreed and disagreed). Respondents also had a similarly varied responses for the statement ‘for IPC to be effective, members of the healthcare team must work within their scope of practice’ (*n* = 49, 71%, strongly agreed and agreed, while *n* = 20, 29%, strongly disagreed and disagreed). A high level of agreement was noted with the hypothesis that training to work on interprofessional teams is critical to the future of medicine (*n* = 60, 86.9%, strongly agreed and agreed).

Overall, using an independent t-test to examine the differences in the means between the group of medical students who had prior exposure to IPE compared to those with no exposure to IPE, demonstrated one statistically significant difference between their attitudes toward the notion that healthcare teams need to trust and respect each other for effective interprofessional collaboration. There were no other significant differences noted.

### 3.3. IPE Definition and Ranking of IPEC Competencies

**Figure 1:**
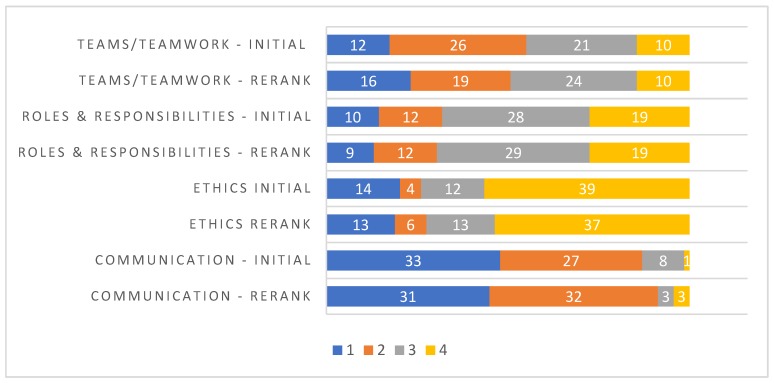
Ranking & re-ranking of IPEC competency. (1 = most important, 4 = least important).

An overwhelming majority of the medical students (*n* = 68, 98.6%) agreed with the WHO’s (2010) definition of IPE. Figure 1 shows the overall initial ranking and re-ranking of the importance of interprofessional education competencies. Responses are stacked as a bar graph, with each bar showing the total number of responses for each of the competencies. The initial ranking and reranking pattern of the competencies were similar, with communication being ranked as the competency of highest importance, and roles and responsibilities being ranked as the least important in both the initial ranking and re-ranking.

### 3.4. Interprofessional Barriers and Opportunities

**Figure 2:**
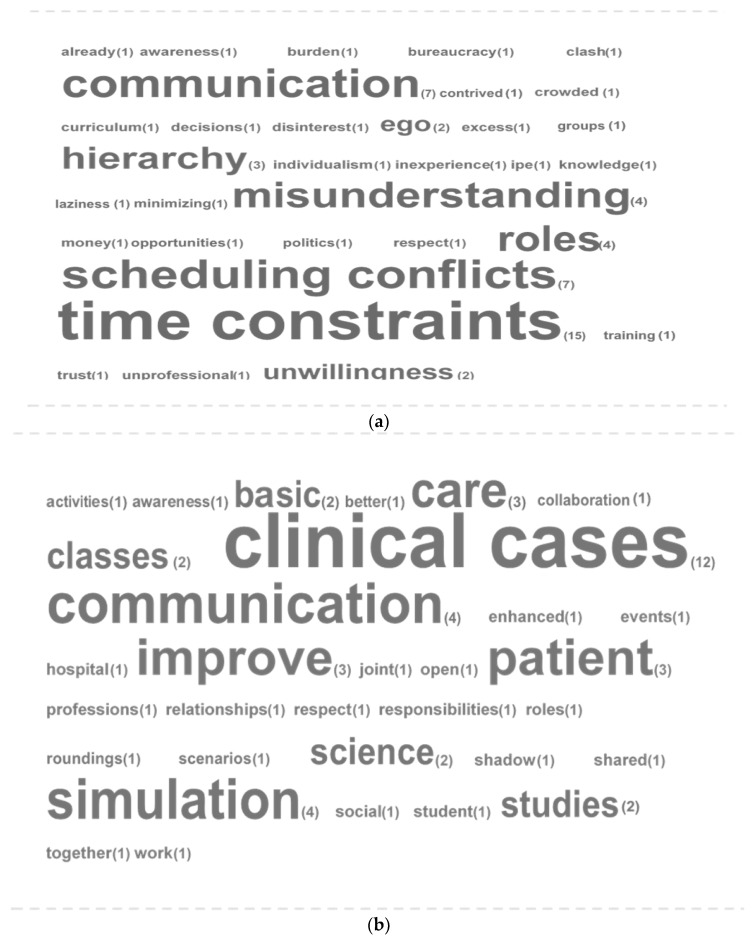
(**a**) TagCrowd analysis of IPE barriers, as perceived by the medical students (**b**) TagCrowd analysis of IPE opportunities, as perceived by the medical students.

A word cloud for IPE barriers is shown in Figure 2a. The word size provides a visual representation of the frequency with which the word was repeated in the survey responses. Actual frequencies are indicated in parentheses adjacent to each word. Time constraints (*n* = 15) emerged as the topmost barrier to IPE, followed by scheduling conflicts (*n* = 7) and communication (*n* = 7). Figure 2b displays word clouds for IPE opportunities. IPE with clinical cases (*n* = 12) was the top opportunity identified by the medical students, followed by simulation (*n* = 4) and communication (*n* = 4).

## 4. Discussion

Interprofessional education (IPE) has increasingly become the impetus behind interprofessional collaboration (IPC) in patient care. Early exposure of students to IPE is required for a positive attitude toward IPC [24]. The results of our study suggest that irrespective of prior exposure to IPE, medical students appreciated the importance of IPE and IPC. This finding is similar to the results reported by Sytsma et al. [25]. In our study, medical students held a firm belief that healthcare teams need to trust and respect each other for effective interprofessional collaboration. They also believed strongly that patients would ultimately benefit when healthcare professionals collaborate to solve patient problems. However, it was interesting to note that some medical students had no opinion on the notion that interprofessional learning enhances their ability to understand clinical problems. This was also evident when ranking the importance of IPEC competencies, where the medical students elected roles and responsibilities as the least important competency during both the initial ranking and re-ranking. This affirms the need for continual emphasis on the various roles performed by other professional disciplines and the necessity to value the unique expertise brought by these professionals in order to provide wholesome patient care.

A large number of medical students ranked communication as the most important IPEC competency in both their initial ranking and subsequent re-ranking. This ranking concurred with previous studies [22,26]. However, a recent study found that communication was ranked as least important among the nutrition program directors [23]. This discrepancy could be due to differences in how students perceive communication when compared to the faculty. Further studies exploring the differences in perception between students and faculty could be valuable.

The majority of the medical students indicated a strong interest in attending IPE coursework with other disciplines, as has been previously suggested by other studies [25]. Among those who had had prior exposure to IPE, many reported being engaged with nursing students. This finding is similar to a previous study by Vernon et al. [22]. In contrast, medical students with IPE had the least engagement with respiratory students. This indicates that most interprofessional experience occurs between medical and nursing students, and least between medical and respiratory therapy students, prompting the need to explore opportunities to integrate medical students with all disciplines.

Time constraints, scheduling conflicts, and communication emerged as the top three barriers to IPE, as perceived by the medical students. The topmost learning method and opportunity in IPE was identified as clinical case studies, followed by simulation and communication. Similar barriers and opportunities have been previously reported [1,27,28].

### Study Limitations

One of the limitations of our survey is the low response rate; this could be attributed to AMSA’s inability to send survey reminders after the initial email. Consequently, the results may not be representative of the medical student population in the United States. Although the roles of IPC and IPE in the improvement of patient health outcomes and healthcare team environments have long been established, low sample sizes constitute a major limitation of many studies [29,30,31]. More effort by researchers is required in creating awareness of the importance of IPE and IPC among healthcare students and professionals, and utilizing innovative means of encouraging potential study participants to respond to study invitations.

Another study limitation may be self-selection bias, whereby students who value IPE were more likely to respond to the survey. In addition, the survey did not capture the locations of the medical schools, such as cities/states. Geographic locations may influence students’ perceptions toward IPE and IPC, and thereby, threaten the generalizability of the presented results.

## 5. Conclusions

Medical practice increasingly relies on interprofessional collaboration to provide high quality patient-centered care. Thus, incorporating IPE early into the medical educational curricula would ensure that medical professionals entering the workforce are well equipped to work in collaborative teams. In general, medical students recognize the significance of interprofessional education and collaboration. Their positive attitude and perceptions toward interprofessional learning and collaboration unlock the potential for medical students to become competent, collaborative healthcare providers in the future.

## Figures and Tables

**Table 1:** Demographic Characteristics of Respondents (*n* = 69).

Variable	*n* = 69	%
**Gender**		
Males	42	61
Females	27	39
**Age in Years**		
>20	1	1.4
20–29	59	85.5
30–39	9	13
**Ethnic Background**		
White, non-Hispanic	48	69.6
Black, non-Hispanic	7	10.1
Hispanic	5	7.2
Asian/non-Pacific Islander, non-Hispanic	7	10.1
Multiple races	2	2.9
**Year in medical school**		
Year 1	17	24.6
Year 2	15	21.7
Year 3	22	31.9
Year 4	15	21.7

**Table 2:** (**a**) Attitudes and perceptions of medical students toward IPE coursework, *n* = 69; (**b**) Attitudes and perceptions of medical students toward IPC in patient care, *n* = 69; (**c**) Attitudes toward Interprofessional Collaboration with healthcare team, *n* = 69.

(**a**)					
	**Strongly Agree**	**Agree**	**Neither**	**Disagree**	**Strongly Disagree**
	***n* (%)**	***n* (%)**	***n* (%)**	***n* (%)**	***n* (%)**
I am interested in coursework with other disciplines because it is important for interprofessional collaboration	31 (44.9)	26 (37.7)	7 (10.1)	4 (5.8)	1 (1.4)
I am interested in attending coursework that includes nurse practitioners and physician assistant students	27 (39.1)	26 (37.7)	8 (11.6)	6 (8.7)	2 (2.9)
I am interested in attending coursework that includes nursing students (not nurse practitioners)	26 (37.7)	35 (50.7)	1 (1.4)	4 (5.8)	3 (4.3)
I am interested in attending coursework that includes allied health professional students	25 (36.2)	32 (46.4)	7 (10.1)	3 (4.3)	2 (2.9)
Interprofessional education helps students think positively about other healthcare professionals	35 (50.7)	22 (31.9)	9 (13)	2 (2.9)	1 (1.4)
Medical students would benefit from working on group projects with students from other health care professions	31 (44.9)	22 (31.9)	9 (13)	4 (5.8)	3 (4.3)
IPE with other disciplines improves interprofessional communication	38 (55.1)	24 (34.8)	6 (8.7)	1 (1.4)	0 (0)
Integrated learning with students in other disciplines helps students to become more effective members of the healthcare team	31 (44.9)	27 (39.1)	6 (8.7)	3 (4.3)	2 (2.9)
Clinical problem solving can only be learned effectively when students are taught within their individual department/ school	10 (14.5)	8 (11.6)	15 (21.7)	27 (39.1)	9 (13)
Interprofessional learning among healthcare students will increase their ability to understand clinical problems	3 (4.3)	12 (17.4)	29 (42)	25 (36.2)	0 (0)
Interprofessional learning will help students to understand their own professional limitations	28 (40.6)	38 (55.1)	3 (4.3)	0 (0)	0 (0)
(**b**)					
	**Strongly Agree**	**Agree**	**Neither**	**Disagree**	**Strongly Disagree**
	***n* (%)**	***n* (%)**	***n* (%)**	***n* (%)**	***n* (%)**
Interprofessional collaboration increases the healthcare team’s ability to understand clinical problems	32 (46.4)	28 (40.6)	6 (8.7)	2 (2.9)	1 (1.4)
Patients receiving interprofessional care are more likely than others to be treated as a whole person	38 (55.1)	18 (26.1)	11 (15.9)	2 (2.9)	0 (0)
The interprofessional approach makes the delivery of patient care more efficient	36 (52.2)	22 (31.9)	10 (14.5)	1 (1.4)	0 (0)
Patients would ultimately benefit if health care professionals worked together to solve patient problems	46 (66.7)	20 (29)	3 (4.3)	0 (0)	0 (0)
The ‘give and take’ among team members helps providers make better patient care decisions	28 (40.6)	32 (46.4)	7 (10.1)	2 (2.9)	0 (0)
Reporting observations to a multi-disciplinary team helps team members better understand the role of other healthcare professionals	32 (46.4)	32 (46.4)	4 (5.8)	1 (1.4)	0 (0)
(**c**)					
	**Strongly Agree**	**Agree**	**Neither**	**Disagree**	**Strongly Disagree**
	***n* (%)**	***n* (%)**	***n* (%)**	***n* (%)**	***n* (%)**
Interprofessional collaboration helps healthcare professionals think positively about the healthcare team	28 (40.6)	29 (42)	9 (13)	2 (2.9)	1 (1.4)
Communication skills are critical for the healthcare team for improved patient outcomes	57 (82.6)	12 (17.4)	0 (0)	0 (0)	0 (0)
Interprofessional collaboration allows healthcare professionals to understand their role limitations	27 (39.1)	36 (52.2)	5 (7.2)	1 (1.4)	0 (0)
For interprofessional collaboration to be effective, the healthcare team needs to trust and respect each other	47 (68.1)	20 (29)	1 (1.4)	1 (1.4)	0 (0)
Team meetings foster communication among members from different professionals or disciplines	38 (55.1)	22 (31.9)	6 (8.7)	3 (4.3)	0 (0)
Working in an interprofessional environment keeps health professionals enthusiastic and interested in their jobs	22 (31.9)	20 (29)	19 (27.5)	5 (7.2)	3 (4.3)
Working in an interprofessional manner requires additional time	20 (29)	16 (23.2)	19 (27.5)	14 (20.3)	0 (0)
For interprofessional collaboration to be effective, members of the healthcare team must work within their scope of practice	26 (37.7)	23 (33.3)	14 (20.3)	6 (8.7)	0 (0)
Training to work on interprofessional teams is critical to the future of medicine	35 (50.7)	25 (36.2)	8 (11.6)	1 (1.4)	0 (0)
